# Antifungal Activity of Siderophore Isolated From *Escherichia coli* Against *Aspergillus nidulans* via Iron-Mediated Oxidative Stress

**DOI:** 10.3389/fmicb.2021.729032

**Published:** 2021-11-03

**Authors:** Azmi Khan, Pratika Singh, Ravinsh Kumar, Sujit Das, Rakesh Kumar Singh, Usha Mina, Ganesh Kumar Agrawal, Randeep Rakwal, Abhijit Sarkar, Amrita Srivastava

**Affiliations:** ^1^Department of Life Science, School of Earth, Biological and Environmental Sciences, Central University of South Bihar, Gaya, India; ^2^Laboratory of Applied Stress Biology, Department of Botany, University of Gour Banga, Malda, India; ^3^Department of Biochemistry, Institute of Science, Banaras Hindu University, Varanasi, India; ^4^School of Environmental Sciences, Jawaharlal Nehru University, New Delhi, India; ^5^Research Laboratory for Biotechnology and Biochemistry (RLABB), Kathmandu, Nepal; ^6^Faculty of Health and Sport Sciences, University of Tsukuba, Tsukuba, Japan

**Keywords:** siderophore, antimicrobials, fungi, oxidative stress, protein modeling

## Abstract

Microorganisms produce various secondary metabolites for growth and survival. During iron stress, they produce secondary metabolites termed siderophores. In the current investigation, antifungal activity of catecholate siderophore produced by *Escherichia coli* has been assessed against *Aspergillus nidulans*. Exogenous application of the bacterial siderophore to fungal cultures resulted in decreased colony size, increased filament length, and changes in hyphal branching pattern. Growth inhibition was accompanied with increased intracellular iron content. Scanning electron microscopy revealed dose-dependent alteration in fungal morphology. Fluorescent staining by propidium iodide revealed cell death in concert with growth inhibition with increasing siderophore concentration. Antioxidative enzyme activity was also compromised with significant increase in catalase activity and decrease in ascorbate peroxidase activity. Siderophore-treated cultures showed increased accumulation of reactive oxygen species as observed by fluorescence microscopy and enhanced membrane damage in terms of malondialdehyde content. Antifungal property might thus be attributed to xenosiderophore-mediated iron uptake leading to cell death. STRING analysis showed interaction of MirB (involved in transport of hydroxamate siderophore) and MirA (involved in transport of catecholate siderophore), confirming the possibility of uptake of iron–xenosiderophore complex through fungal transporters. MirA structure was modeled and validated with 95% residues occurring in the allowed region. *In silico* analysis revealed MirA–Enterobactin–Fe^3+^ complex formation. Thus, the present study reveals a promising antifungal agent in the form of catecholate siderophore and supports involvement of MirA fungal receptors in xenosiderophore uptake.

## Introduction

Microbes are known to produce different types of secondary metabolites for their growth and survival. The Gram-negative bacteria *Escherichia coli* produces low-molecular-weight secondary metabolites termed siderophores under iron-stressed conditions (Neilands, 1995). In the environment, iron predominantly exists in its oxyhydroxide form (Fe^3+^) as iron readily oxidizes to insoluble oxyhydroxide polymers at biological pH and aerobic conditions (Paul and Dubey, 2015). This makes them inaccessible for microbial metabolic processes since iron can only be utilized in its ferrous state (Fe^2+^). Microbes have evolved various methods to acquire iron. For example, iron uptake in *Aspergillus fumigatus* occurs *via* reductive iron assimilation pathway that involves an *ftr* A permease and in *Pseudomonas* sp. *via* the FpvA/FpvR/PvdS signaling pathway (Lamont et al., 2002; Schrettl et al., 2004). However, most microbes have developed siderophore-mediated iron acquisition system to scavenge iron from their surrounding (Schwyn and Neilands, 1987; Neilands, 1995). Siderophores are non-ribosomal peptides classified into hydroxamate, catecholate, carboxylate, and mixed types (Khan et al., 2018).

*Aspergillus* spp. are causative agents of pneumonia and invasive disseminated disease in immunocompromised patients (Segal et al., 1998). Effect of secondary metabolites of plants and microorganisms has been extensively studied. Siderophores, partially purified from different sources such as *Acinetobacter colcoaceticus* and *Pseudomonas aeruginosa* JAS25, among others, have been studied for their antifungal activity against phytopathogens like *Fusarium oxysporum, Fusarium udum*, and *Aspergillus niger*; however, the mechanism behind the observed effect has not been assessed extensively (Maindad et al., 2014; Sulochana et al., 2014). The antifungal activity of siderophores isolated and purified from *E. coli* and the mechanism behind the proposed effect has not been assessed so far. There is a need for potent antifungal agents isolated from natural sources other than synthetic ones. In the present study, antifungal activity of purified catecholate siderophores of *E. coli* has been assessed. The results indicated enhanced iron accumulation in *Aspergillus nidulans* in the presence of catecholate siderophore. To the best of our knowledge, this is the first report of xenosiderophore induced oxidative stress occurring due to iron excess. Xenosiderophore uptake is of common occurrence among microbes. Several bacteria and fungi are known to possess receptors for siderophores other than the ones they produce (Haas et al., 2003). Accumulation of iron has been associated with increase in reactive oxygen species (ROS) followed by lipid peroxidation leading to membrane damage and cell death termed ferroptosis (Dixon et al., 2012). Here, we report that the catecholate type of siderophore inhibits fungal growth in a dose-dependent manner possibly through involvement of fungal transporter for siderophore.

## Materials and Methods

### Culture of Experimental Organisms

Cultures of *E. coli* BL21 DE3 were maintained in Luria Bertani (LB) broth and chemically defined low iron media (CDLIM; K_2_SO_4_ 2 g L^–1^, K_2_HPO_4_ 3 g L^–1^, NaCl 1 g L^–1^, NH_4_Cl 5 g L^–1^, MgSO_4_ 80 mg L^–1^, ZnSO_4_ 5.5 mg L^–1^, CuSO_4_ 20 μg L^–1^, MnSO_4_ 0.9 mg L^–1^, Thiamine HCl 2 mg L^–1^, Glycerol 25 ml L^–1^, and 1 ml of 0.5 M CaCl_2_) at 37°C for 24 h. *A. nidulans* (NCCPF no. 850001) was grown in rich Yeast Extract Peptone Dextrose media (YEPD) at 37°C for 48 h. For preparation of spore suspension, *A. nidulans* was first grown on YEPD agar plates and harvested using phosphate buffer saline (PBS) containing 0.05% tween-20.

### Qualitative and Quantitative Analysis of Siderophore

Presence of catecholate siderophore was confirmed by Arnow assay (Arnow, 1937). Quantification of siderophore was also done by Arnow assay. For this purpose, 1 ml of sample is mixed with 1 ml of HCl (0.5 N), 1 ml of nitrite molybdate (10 g of sodium nitrite and sodium molybdate in 100 ml of distilled water), and 1 ml of NaOH (1 M) in a sequential manner. From this mixture, 300 μl was pipetted out and added to separate wells in a 96-well plate and absorbance was then recorded at 490 nm using a microplate reader. A standard curve was also prepared using varying concentrations of 2,3-Dihydroxybenzoic acid (2,3-DHBA) as standard by performing Arnow assay as explained above. Siderophore concentration in unknown sample was calculated using the line equation obtained from the standard curve and expressed in terms of μg/ml equivalent of 2,3-DHBA (Sridevi et al., 2008).

### Siderophore Purification

Two bed volume of supernatant acidified to pH 2 ± 0.2 was loaded on the XAD-2 resin column. Elution was performed after 4 h using methanol at a flow rate of 1.5 ml/min. No external pressure was applied and the column was allowed to flow down by gravity at room temperature. Sequential fractions were collected and catecholate positive fractions were pooled together. Methanol was then evaporated using a rotary evaporator (40°C; 110 mbar pressure; Khan et al., 2020). Samples were further purified by size exclusion chromatography using Sephadex LH20 resin. Two percent of the bed volume of XAD-purified and rotary evaporated fractions were loaded in the column packed with Sephadex LH20 resin and immediately elution was started to remove impurities at 1.5 ml/min of flow rate under the action of gravity again. Methanol was then added to the column and elution was carried out at same flow rate to collect sequential fractions. All the fractions were again tested for the presence of catecholate, positive fractions were pooled together, and again rotary evaporated under the same conditions as mentioned above.

### Reverse-Phase High-Performance Liquid Chromatography Analysis

The purified sample was analyzed by Reverse-Phase High-Performance Liquid Chromatography (RP-HPLC) using C18 hydrophobic column on Shimadzu HPLC system with UV-Vis detector. HPLC grade filtered methanol was used as mobile phase. 2,3-Dihydroxybenzoic acid 99% (Sigma Aldrich) was obtained in powdered form and was used as standard. It was first dissolved in HPLC grade methanol at a concentration of 50 μg/ml and then filtered using a syringe filter of pore size 0.22 μM. LH purified sample was also filtered similarly. The column (30 × 2.1 mm) was first washed using filtered HPLC grade methanol for half an hour. For the analysis, 20 μl of standard as well as sample was used as injection volume. A flow rate of 1.5 ml/min was set. Peaks were analyzed at 227 nm and retention time was recorded.

### Antifungal and Iron Supplement Assay

Four sets of YEPD broth were prepared with 18, 36, 44, and 54 μg/ml of catecholate siderophore, inoculated with 1 ml of spore suspension containing 10^6^ fungal spores and incubated under standard conditions. YEPD agar plates were prepared with similar catecholate concentrations (except 54 μg/ml) in three sets; without supplemented iron, with supplement of 2 and 4 mM FeCl_3_. Plates were inoculated with fungal spores and observed for 48 h after incubation. Total biomass of fungal culture with and without siderophore treatment was measured with modifications in protocol of Granade et al. (1985). For this purpose, fungal cultures from YEPD broths were filtered using Whatman filter paper no. 1. Mycelia thus obtained were then washed thoroughly in PBS (pH 7.4). The washed mycelia were then dried in oven overnight at 60°C and weighed. For measurement of filament length, 0.25 g of fungal filaments grown on each YEPD agar plate with varying concentrations of siderophore were stained with lactophenol cotton blue (LPCB; Leck, 1999) and observed at 40× magnification under a Nikon Eclipse TiU inverted microscope. Filament length was measured using Nikon NIS element software.

### Intracellular Iron Content

For determination of intracellular iron content, acid digestion was carried out according to the method given by EPA (1996); 0.25 g fresh weight from 48 h grown samples were left overnight in 20 ml of HNO_3_ and 3 ml of distilled water. Samples were heated at 105°C for 2.5 h. After cooling, 10 ml of 35% HCl was added and again heated at 105°C. Three milliliters of 30% H_2_O_2_ was added after cooling and reheated at 105°C. This solution was filtered through Whatman filter paper no. 1. Samples were diluted in 0.2 M acetic acid and 0.2 M sodium acetate buffer. Fe^2+^ ion was measured by atomic absorption spectrometry (AAS; Perkin Elmer AAnalyst 800) using ferrous ammonium sulfate as standard.

### Light and Scanning Electron Microscopy

Filaments grown in YEPD broth were stained with LPCB and observed at 100× magnification under an Olympus CX31 optical microscope. Filament length was measured using Nikon NIS element software.

For scanning electron microscopic analysis, samples were prepared according to Singh et al. (2021) with slight modification in concentration of glutaraldehyde. Fungal mat from 48 h plate culture (treated and untreated) was removed from plate and washed thrice in PBS (pH 7.4) thoroughly. Samples were weighed and 0.25 g from each sample was then fixed in 3% glutaraldehyde. For this, 50% glutaraldehyde solution was diluted to 3% with 0.1 M sodium phosphate buffer (pH 7.2). Fixation was done overnight and then further dehydration was performed. For dehydration, sample was sequentially dipped for 1 min each in different gradients of ethanol, i.e., 10%, 30%, 50%, 70%, 90%, and absolute ethanol. Surface characterization was then carried out in high vacuum mode using a Hitachi S-3400N scanning electron microscope.

### Propidium Iodide Staining

Fungal cultures after 48 h of treatment were scraped from culture plates and weighed, and 100 mg of each sample was then washed separately and thoroughly in PBS (pH 7.4). Sample was then recovered by subjecting them to centrifugation at 5,000 *g* for 5 min. Cultures were resuspended in 1 ml of PBS in different wells of a labeled 12-well plate. To each well, propidium iodide (PI) was added at a final working concentration of 10 μg/ml (stock—1 mg/ml in distilled water; Fernández-Miranda et al., 2017). The plates were kept at 37°C for 25 min with gentle agitation. After incubation, cultures were washed twice in PBS and finally resuspended in PBS. Fluorescence was observed with excitation at 493 nm and emission at 636 nm using a Nikon Eclipse TiU inverted microscope. Mean fluorescence intensity was then measured using ImageJ 1.52a software.

### 2′,7′-Dichlorofluorescin Diacetate Staining

Treated and untreated fungal cultures were scraped and weighed, and 100 mg from each sample was separately washed twice in PBS. Dichlorofluorescin Diacetate (DCFDA) stock was prepared in DMSO and washed fungal cultures were treated with it at a working concentration of 50 μg/ml DCFDA in PBS and incubated overnight at 37°C with gentle agitation. After incubation, fluorescence was observed with excitation at 485 nm and emission at 535 nm using a Nikon Eclipse TiU inverted microscope. Mean fluorescence intensity was then calculated using ImageJ 1.52a software.

### Antioxidative Enzyme Assays

Whole cell protein was extracted by urea buffer extraction method (Osherov and May, 1998). Fungal cultures were freeze dried and crushed manually. Lyophilized sample (100 mg) was mixed with urea lysis buffer (1% SDS, 7 M urea, 25 mM Tris HCl, 1 mM EDTA, and 0.7 M β-mercaptoethanol), boiled at 95°C for 2 min, vortexed, and again boiled for 1 min. The slurry was centrifuged for 40 min at 13,500 *g* at 4°C. Supernatant was used for enzymatic analyses as mentioned below. Total protein content was estimated by Bradford assay (Bradford, 1976).

#### Superoxide Dismutase Assay

Three milliliters of reaction mix [100 mM phosphate buffer, 0.2 M methionine, 2.25 mM nitro blue tetrazolium chloride (NBT), 30 mM EDTA, 60 μM Riboflavin, and 1.5 M sodium carbonate] was added to 100 μl of enzyme extract and placed under light for 10 min followed by transfer to dark. SOD is expressed as unit of activity/μg protein after measuring A_560_ (Beauchamp and Fridovich, 1971). One unit of SOD is equivalent to amount of enzyme causing 50% reduction in inhibition of NBT.

#### Catalase Assay

To 0.05 ml of enzyme extract, 1.5 ml of PBS and 950 μl of distilled water was added. To start the reaction, 0.5 ml of H_2_O_2_ mix (775 μl of H_2_O_2_ in 100 ml distilled water) was added. Decrease in the A_240_ of H_2_O_2_ was recorded in the presence of enzyme extract following the protocol of Aebi (1984). CAT activity was expressed as unit/min/μg of protein.

#### Ascorbate Peroxidase Assay

H_2_O_2_ (100 μl) was added to the 2-ml reaction mix (50 mM phosphate buffer, 1 mM EDTA, 5 mM ascorbate, and 100 μl of enzyme extract). Reduction in A_290_ was recorded for 3 min. Enzyme activity was expressed as units/min/mg protein using 2.8 mM^–1^cm^–1^ as extinction coefficient.

### Lipid Peroxidation

Mycelia (0.5 g) were homogenized with 0.1% trichloroacetic acid (TCA) and centrifuged at 21,000 *g* for 15 min. Four milliliters of 0.5% TBA (w/v) in 20% TCA (v/v) was added to 1 ml of supernatant and boiled at 95°C for 30 min. Cooled mixture was centrifuged at 150 *g* for 10 min. Absorbance of supernatant was taken at 532 and 600 nm (Heath and Packer, 1968).

### STRING Analysis

For catecholate siderophore transport in *A. nidulans*, expected functional partners were analyzed and confirmed using STRING database server^1^ (Khan et al., 2020).

### Molecular Modeling and Validation of MirA Receptor

The FASTA sequence of MirA was retrieved from UniprotKB (Uniprot Id- Q8X1Z7) and submitted to SWISS-MODEL in automated modeling mode (Waterhouse et al., 2018). The generated model was evaluated using MOLprobity server (Williams et al., 2018) for Ramachandran plot analysis and ProSA server (Wiederstein and Sippl, 2007) to generate *Z*-score.

### Ligand (Enterobactin-Fe) Preparation

2D structures of Fe and Enterobactin (Ent; PubChem CID- 34231) were retrieved in spatial data file (sdf) format from PubChem. 3D structures were generated using Cactus server.^2^ Ent was subjected to the LigPrep program in Maestro 10.6 for generation of its ionization states (pH 7 ± 2) and possible tautomers with Epik and OPLS3 force field.

### Docking and Interaction Analysis

Docking of Ent and Fe was performed using Hex 8.0.0 software and the complex generated was further docked with MirA using AutoDock Vina (Trott and Olson, 2010). A grid box of 85, 75, 115 (X, Y, and Z) was used after adding hydrogen, Gastreiger, and Kollman charges. Visualization and interaction analysis were done using UCSF chimera 1.14 and LigPlot^+^ v2.2.

### Statistical Analysis

GraphPad prism 5.01 was used to compute standard error and significance of quantitative changes under different treatment conditions in three replicates by one-way ANOVA and Tukey’s post-test. Data are expressed as mean ± SE and differences were considered highly significant at *p* ≤ 0.001, very significant at *p* ≤ 0.01, and significant at *p* ≤ 0.05.

## Results

### Purity Analysis by High-Performance Liquid Chromatography

**FIGURE 1:**
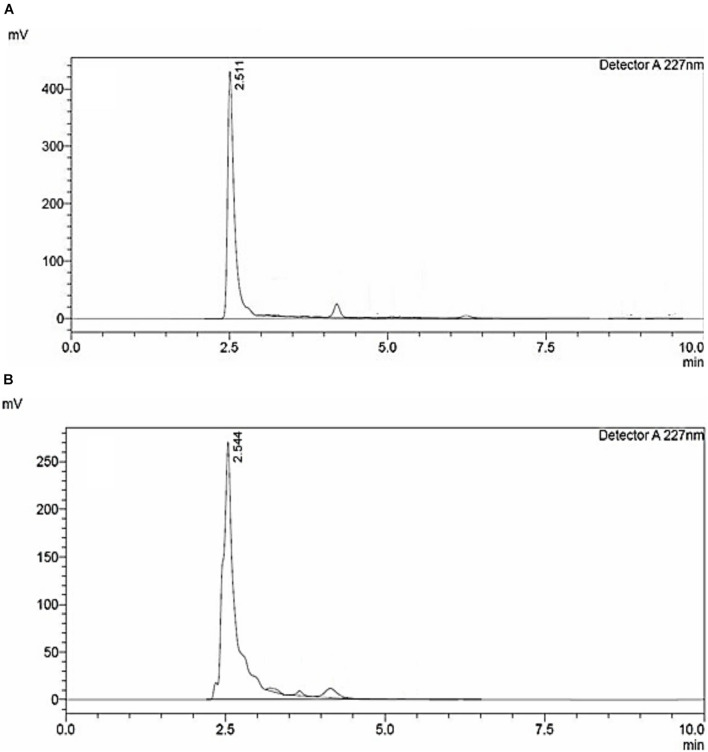
HPLC chromatogram of **(A)** 2,3-Dihydroxybenzoic acid; standard. **(B)** Catecholate siderophore purified from *E. coli* culture.

High-performance liquid chromatography analysis of siderophores isolated and purified from *E. coli* culture showed similar retention time as seen in standard (Figure 1A). A major peak was revealed at 227 nm at a retention time of 2.544 min (Figure 1B), close to the peak observed in standard having a retention time of 2.511 confirming the purity of catecholate siderophore samples. The purified siderophore was thus tested for its antifungal efficacy.

### Antifungal Activity of Enterobactin

**FIGURE 2:**
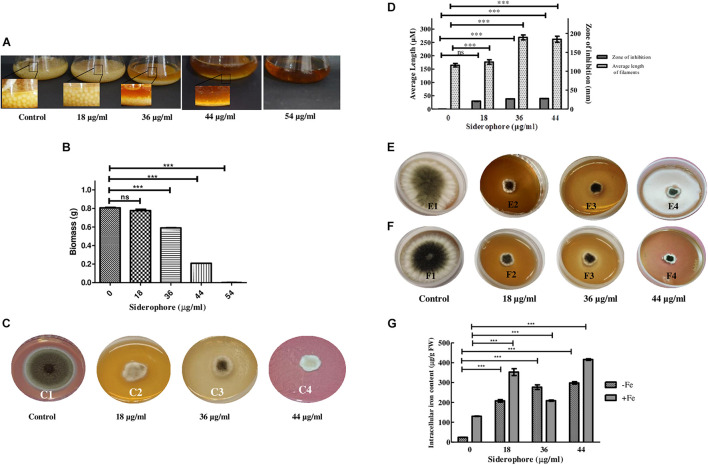
Effect of varying siderophore concentrations after 48 h on **(A)** colony of *A. nidulans* in YEPD culture (Inset—colony size). **(B)** Biomass of *A. nidulans* in YEPD culture. **(C)** Growth of *A. nidulans* on YEPD solid media under varying concentrations of siderophore: 1—no siderophore, 2—18 μg/ml, 3—36 μg/ml, 4—44 μg/ml. **(D)** Average filament length and zone of inhibition at different siderophore concentrations without iron supplement. **(E)** Growth of *A. nidulans* on YEPD media with 2 mM FeCl_3_ supplement under varying concentrations of siderophore: 1—no siderophore, 2—18 μg/ml, 3—36 μg/ml, 4—44 μg/ml. **(F)** Growth of *A. nidulans* on YEPD media with 4 mM FeCl_3_ supplement under varying concentrations of siderophore: 1—no siderophore, 2—18 μg/ml, 3—36 μg/ml, 4—44 μg/ml. **(G)** Intracellular iron content of *A. nidulans* under iron deplete and 4 mM iron supplement at different siderophore treatments (ns—non-significant, ***p ≤ 0.001—highly significant).

After 48 h of inoculation, the colony size of *A. nidulans* grown in liquid YEPD media decreased with increasing concentration of catecholate siderophore. Growth with reduced colony size was observed up to 44 μg/ml with complete inhibition at 54 μg/ml, which was considered as the minimum inhibitory concentration of siderophore for the fungus (MIC; Figure 2A). Total biomass also decreased in a dose-dependent manner. Excluding MIC, the lowest biomass (0.208 g) was observed in cultures treated with 44 μg/ml of siderophore, which was 64.65% less than the biomass of untreated control (0.8063 g; Figure 2B).

Similar results were obtained in plate assay (Figure 2C). Growth was observed after 48 h of incubation, which was, however, less than control. The largest zone of inhibition of 3.96 cm was observed at 44 μg/ml (Figure 2D). Length of filaments increased at lower concentrations of catecholate siderophore, i.e., 18 and 36 μg/ml, and remained the same as the latter at 44 μg/ml (Figure 2D). Confirmation of growth inhibition was followed by morphological and biochemical analyses to deduce the basis of the effect of siderophore on *A. nidulans*.

### Iron Supplement Assay and Intracellular Iron Content

Presence of siderophore in the media was expected to alter the iron status of the fungus. To assess whether the observed growth inhibition involved extracellular iron chelation by siderophore, rendering iron unavailable to the test organism, an iron supplement assay was performed. When cultures were subjected simultaneously to catecholate siderophore and additionally supplemented iron, growth was not restored at 2 mM as well as 4 mM of iron (Figures 2E,F). Assessment of intracellular iron content revealed that exposure to increasing siderophore treatment led to increased accumulation of iron within fungal cells. Without iron supplementation, maximum iron content of 298.32 μg g^–1^ FW was observed at 44 μg/ml siderophore treatment that was 12-fold higher than iron content of control (23.9 μg g^–1^ FW). Thus, presence of siderophore led to enhanced intracellular iron accumulation. When supplemented with 4 mM of FeCl_3_, intracellular iron content at 44 μg/ml intensified further and 416.3 μg Fe^2+^ g^–1^ FW was observed (Figure 2G).

### Morphological Analysis by Microscopy

#### Light Microscopy

**FIGURE 3:**
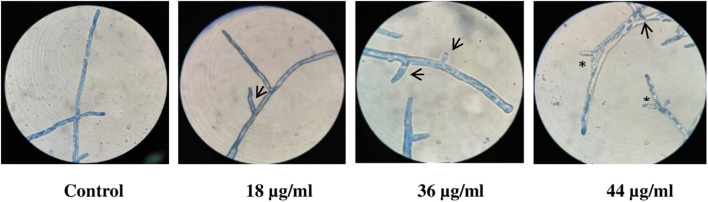
LPCB stained fungal hyphae treated with varying concentrations of siderophore: 1—no siderophore, 2—18 μg/ml, 3—36 μg/ml, 4—44 μg/ml (asterisk—apical branching; arrow—lateral branching; magnification—100×).

Microscopic observation of LPCB-stained fungal cultures revealed more lateral branching with increasing concentrations of catecholate siderophore. Apical branching in cultures treated with 44 μg/ml was observed to be enhanced as compared to control (Figure 3).

#### Scanning Electron Microscopy

**FIGURE 4:**
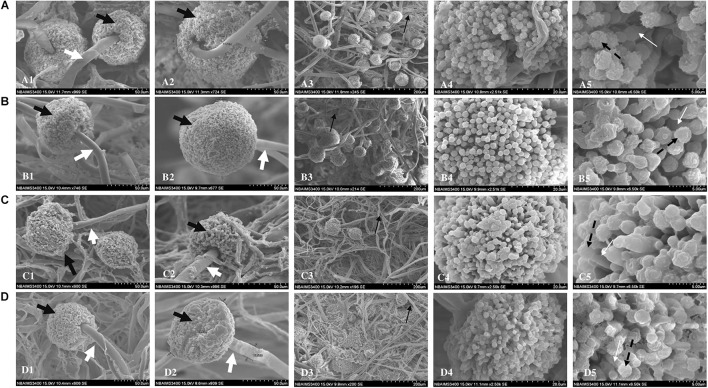
Scanning electron micrographs of *A. nidulans* **(A)** without siderophore treatment: (1–2) Conidiophores and conidial heads; (3) Hyphae; (4) Conidial head (magnification—20 μm); (5) magnified conidia and phialides (5 μm); **(B)** treated with 18 μg/ml of siderophore: (1–2) Conidiophores and conidial heads; (3) Hyphae; (4) Conidial head (magnification—20 μm); (5) magnified conidia and phialides (5 μm); **(C)** treated with 36 μg/ml of siderophore: (1–2) Conidiophores and conidial heads; (3) Hyphae; (4) Conidial head (magnification—20 μm); (5) magnified conidia and phialides (5 μm); **(D)** treated with 44 μg/ml of siderophore: (1–2) Conidiophores and conidial heads; (3) Hyphae; (4) Conidial head (magnification—20 μm); (5) magnified conidia and phialides (5 μm); Black bold arrow—conidial head; White bold arrow—conidiophores; Black thin arrow—hyphae; Black dashed arrow—conidia; White thin arrow—phialides.

Scanning electron microscopy revealed morphological changes in fungal structures in a dose-dependent manner. While control samples showed normal morphological features (Figure 4A), samples treated with siderophore showed considerable damage to morphology. At 18 μg/ml, not much distortion was observed while morphological changes were observed at 36 μg/ml (Figures 4B,C). Maximum damage in the form of collapsed hyphae was observed at 44 μg/ml of siderophore as compared to control (Figure 4D). Hyphal structures appeared more flattened and more branching was evident with increasing siderophore concentration. The typical morphology of phialides appeared more damaged and distorted in siderophore-treated culture as compared to control. Furthermore, the diameter of conidiophores as well as number of conidial heads reduced with increasing siderophore concentration whereby minimum number of conidiophores was observed at 44 μg/ml. Thus, overall damage to morphology of *A. nidulans* was observed under siderophore treatment.

### Propidium Iodide Staining

**FIGURE 5:**
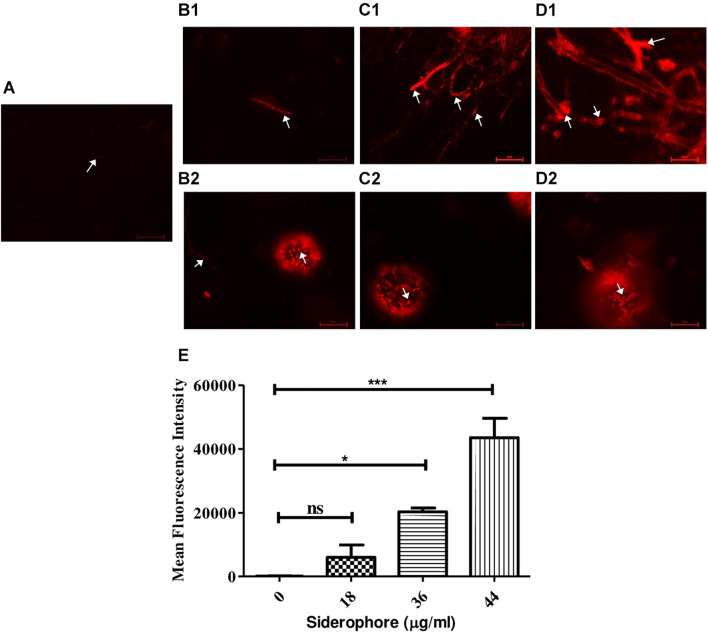
Microphotographs of *A. nidulans* under various siderophore treatments after staining with propidium iodide (PI). **(A)** Control; **(B1)** Hyphae and **(B2)** conidial head of cultures treated with 18 μg/ml of siderophore; **(C1)** Hyphae and **(C2)** conidial head of cultures treated with 36 μg/ml of siderophore; **(D1)** Hyphae and **(D2)** conidial head of cultures treated with 44 μg/ml of siderophore (arrows indicating cellular damage). **(E)** Mean fluorescent intensities from PI staining (ns—non-significant, ***p ≤ 0.001—highly significant, and *p ≤ 0.05—significant).

Propidium iodide staining was performed to assess cellular damage to the fungal cells due to siderophore treatment. It was observed that after 48 h of siderophore treatment, fluorescence intensity from PI staining increased in a dose-dependent manner (Figure 5). While untreated cultures showed negligible amount of fluorescence confined to spores only (Figure 5A), in siderophore-treated cultures, damage to hyphae as well as conidial heads was evident in the form of intense PI fluorescence (Figures 5B–D). 48.02−, 162.89−, and 349.41-fold increase in fluorescent intensity was recorded at 18, 36, and 44 μg/ml of siderophore, respectively, as compared to the untreated control (Figure 5E).

### Reactive Oxygen Species Accumulation, Antioxidative Enzyme Activity, and Membrane Damage

**FIGURE 6:**
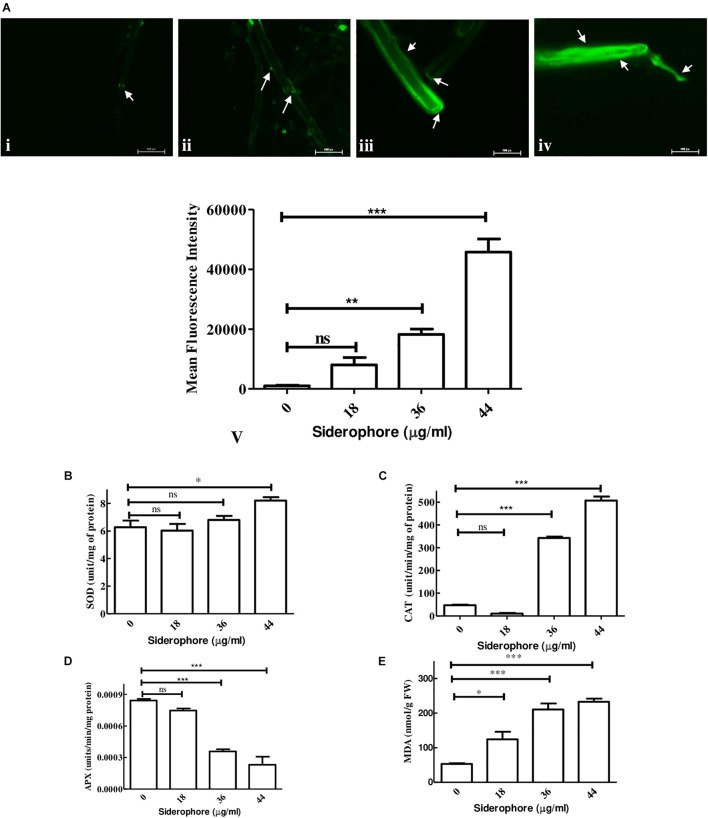
Effect of varying concentrations of catecholate siderophore on **(A)** ROS accumulation visualized by DCFDA staining (arrows indicate representative sites of ROS accumulation): i—control; ii—18 μg/ml; iii—36 μg/ml; iv—44 μg/ml; v—mean fluorescent intensities from DCFDA staining. **(B)** Superoxide dismutase activity. **(C)** Catalase activity. **(D)** Ascorbate peroxidase activity. **(E)** MDA content in *A. nidulans* (ns—non-significant, ^∗∗∗^*p* ≤ 0.001—highly significant, ^∗∗^*p* ≤ 0.01—very significant, and ^∗^*p* ≤ 0.05—significant).

Analysis of possible oxidative stress in siderophore-treated cultures was carried out by measuring the level of ROS production, antioxidative enzymes, and degree of membrane damage. In DCFDA staining, least fluorescence was observed in untreated cultures while the intensity increased at each concentration of siderophore as compared to control (Figure 6Ai–iv). In comparison to untreated control, 7. 86−, 17. 80−, and 44.71-fold increase in DCFDA fluorescence was recorded in cultures treated with 18, 36, and 44 μg/ml of siderophore (Figure 6A-v). No significant change was observed in SOD activity after treatment of fungal cells with 18 and 36 μg/ml of siderophore. Significant change (*p* < 0.01) in SOD activity was observed at 44 μg/ml with an increase of 30.78% as compared to control (Figure 6B). Lowest CAT activity was observed upon treatment with 18 μg/ml, 78.28% lesser than control. A highly significant increase (*p* < 0.0001) of 7.29 and 10.80 times in CAT activity as compared to control was observed in 36 and 44 μg/ml treated cultures, respectively (Figure 6C). APX activity showed a highly significant (*p* < 0.0001) decrease of 57.44% at 36 μg/ml as compared to control. Lowest APX activity was observed at 44 μg/ml, i.e., 72.48% lesser than control (Figure 6D). Maximum MDA content was observed in cultures treated with highest siderophore concentration (44 μg/ml). As compared to control, a significant (*p* < 0.01) increase of 2.35 times and a highly significant (*p* < 0.0001) increase of 3.97 and 4.39 times was recorded at 18, 36, and 44 μg/ml of siderophore (Figure 6E).

### STRING Analysis

**FIGURE 7:**
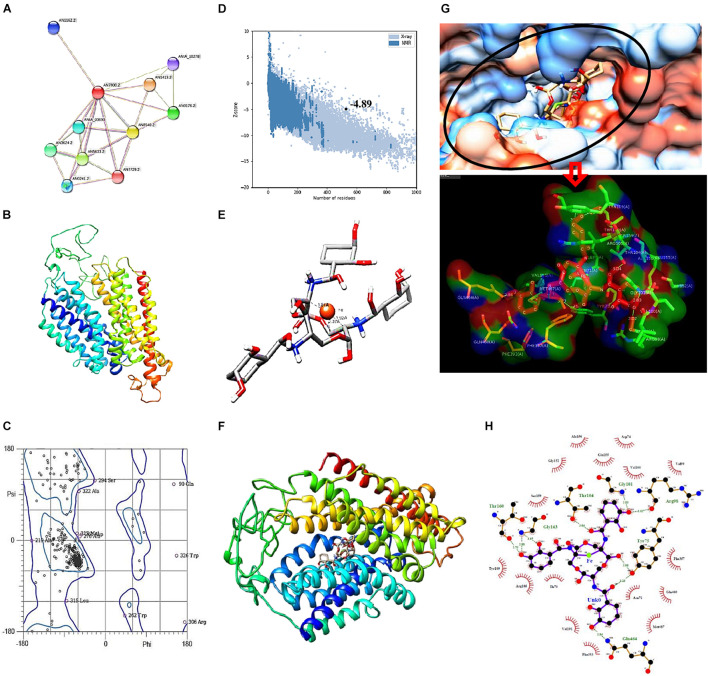
**(A)** STRING analysis showing protein–protein interaction for siderophore import in *A*. *nidulans*. Green lines connect proteins that are associated by recurring neighborhood, blue connections are inferred by phylogenetic co-occurrence, and red lines indicate gene-fusion events; line thickness is a rough indicator for the strength of the association. Number of nodes: 11, number of edges: 25, average node degree: 4.55, average local clustering coefficient: 0.776. **(B)** Structure of MirA generated by SWISS-MODEL. **(C)** Ramachandran plot of MirA model. **(D)** *Z*-score plot generated by ProSA web server for MirA model. **(E)** Docked complex of Enterobactin (Stick) and Iron (Ball) with distance measurement between Fe and C. **(F)** Docked complex of MirA with Ent–Fe complex. **(G)** Binding pocket of MirA as visualized in hydrophobicity surface mode, inset: interacting residues of MirA (surface) with Ent–Fe complex (stick). **(H)** Interaction plot generated by Ligplot+; green dotted lines represent hydrogen bonds with distance.

Growth inhibition associated with altered ROS status and increased intracellular iron content indicated enhanced uptake of iron in the presence of catecholate siderophore. *In silico* confirmation of the possibility of iron acquisition *via* uptake of the Fe^3+^–enterobactin complex was made by looking for proteins related to siderophore uptake and docking the Fe^3+^–enterobactin complex with known fungal receptors. STRING analysis confirmed MirA (AN7800.2; Siderophore iron transporter) to be enterobactin transporter (known catecholate siderophore of *E. coli*) in *A. nidulans.* It interacts with MirB (AN8540.2; transporter for TAFC) and both MirA and MirB have transmembrane transport activity. These two, together with SreA (AN0176.2; Siderophore biosynthesis repressor), have a role in maintaining cellular iron homeostasis (Figure 7A).

### Molecular Modeling and Validation of MirA Receptor Protein

Fungal receptor, MirA, was modeled so as to assess its complex forming ability with the siderophore–Fe^3+^ complex. The model consists of 14 major alpha helices connected by loops and without β-sheets (Figure 7B). Analysis of Ramachandran plot for this structure revealed 95.6% residues in allowed regions while only 4.39% were outliers (Figure 7C). The overall assessment of model quality using ProSA web server gave a *Z*-score of −4.89 (Figure 7D).

### Ligand Preparation and Docking

Analysis of stable complex formation between MirA receptor and the ligand Ent–Fe required optimization of enterobactin’s structure. Out of 54 poses of enterobactin, best pose on the basis of Epik state penalty was selected (0.7315 kcal mol^–1^; lowest state penalty represents energetically more stable structure) and further employed for docking with the fungal receptor. The Ent–Fe complex showed an *E*_total_ value of −52.12. A distance of 1.81, 1.92, and 2.30 Å was noted between Fe and carbon atoms at positions 22, 19, and 20 (Figure 7E). AutoDock Vina generated a total of nine conformations by docking MirA with the Ent–Fe complex. Conformation with best binding affinity (−10.5 kcal mol^–1^) was selected (Figure 7F). Interaction analysis of binding pocket revealed presence of polar (Thr-104, Gln-464, and Thr-160), non-polar (Gly-101 and Gly-163), charged (Arg-98), and an aromatic (Tyr-75) amino acid residue involved in hydrogen bonding with the Ent–Fe complex. Few more residues (Gly-152, Val-100, Val-199, Phe-357, Gln-460, Asn-71, Met-487, Phe-393, Val-191, Ile-70, Arg-105, Ser-159, Tyr-109, Ala-156, and Glu-155) were involved in forming hydrophobic contact with the Ent–Fe complex (Figures 7G,H).

## Discussion

Siderophores, the secondary metabolites of various microorganisms secreted under iron stress, have been a subject of interest for research for decades. Siderophores not only are useful for iron acquisition in their producers but also confer antimicrobial properties providing competitive advantage over non-producers (Lamb, 2015).

Among all other secondary metabolites, antifungal, antibacterial, and algicidal activities of siderophores produced by *Pseudomonas, Penicillium*, and *Stenotrophomonas* are well validated (Liu et al., 2014; Sulochana et al., 2014; Chowdappa et al., 2020). While siderophore from bacterial origin affected the spore germination and mycelial growth in turn inhibiting growth of fungal phytopathogen *Fusarium* spp. and *Aspergillus*, the siderophore of fungal origin inhibited bacterial growth of *Ralstonia solanacearum* and *Xanthomonas oryzae* pv. Reduced growth and biomass of *A. nidulans* with increasing concentration of *E. coli* catecholate siderophore indicates a stressful environment for the fungus. Bearing conidia is an attempt of fungi to overcome stressful condition by dispersal though spores (Skromne et al., 1995). Stresses such as nutrient depletion, osmotic insult, or other oxidative are known to increase conidiophore development in *Aspergillus* sp. by increasing mycelia formation (Adams et al., 1998; Gavrias et al., 2001). The increase in fragment length upon treatment with siderophore suggests activation of such adaptive strategies to combat iron stress. In *A. fumigatus*, reductive iron assimilation occurs that partially compensates for lack of siderophores (Schrettl et al., 2004). However, this pathway has not been reported up till now in *A. nidulans*, suggesting considerable dependence of the organism on siderophore for iron acquisition. Thus, it was speculated that the iron stress created by bacterial catecholate siderophore could not be compensated by the fungus in the absence of an alternative mechanism for iron acquisition.

Branching of hyphae augments assimilation of nutrients. Abnormal accumulation of exocytic vesicles is responsible for apical branching at hyphal tip enabling growth under certain stress conditions (Watters and Griffiths, 2001). This results in breaking the phenomenon of apical dominance causing a disordered growth pattern affecting colony development (Semighini and Harris, 2008). Increase in apical branching pattern at higher catecholate siderophore concentration observed in light microscopy suggests activation of a similar kind of a stress response in *A. nidulans.* Flattened and ruptured hyphae, clearly visible in SEM analysis, might be due to catecholate siderophore treatment that weakens cell walls leading to release of cytoplasmic content upon exposure to vacuum pressure applied during microscopy. A similar effect was observed upon treatment with crude lysate isolated from *Bacillus* sp. leading to shrunken and disrupted hyphal walls in *Aspergillus* spp. (Singh et al., 2015). Oxidative stress-induced morphological changes were also observed in *A. nidulans* treated with cellulose synthase inhibitor dichlobenil (García-Angulo et al., 2009; Guerriero et al., 2013) and in copper-treated *A. niger* isolate UCP/WFCC 126 (Cavalcanti et al., 2015). Such reports also indicated the possibility of oxidative stress as a reason for morphological damage in *A. nidulans* induced by catecholate-mediated iron accumulation. Production of conidia for the purpose of asexual reproduction in *A. nidulans* requires formation of conidiophores governed by various genetic and metabolic regulations (Gavrias et al., 2001). While mycelial fragments appeared to increase in length as observed in light microscopy, reduction in number of conidiophores and distortion in structure of phialides indicates failure of complete adaptive response and inhibition of progression of fungal development by means of asexual reproduction with increasing catecholate treatment. Similar inhibition in conidiophore development has been earlier described in *A. fumigatus* treated with cytosolic antifungal proteins of *E. coli* DH5α (Balhara et al., 2014).

Propidium iodide, a membrane-impermeable dye, stains dead cells with compromised membrane by entering into cells and binding with nucleic acid and gives a red fluorescence upon excitation with green light at 493 nm (Arndt-Jovin and Jovin, 1989). The fluorescence intensity thus produced is proportional to membrane damage and cell death. Increasing intensity of PI with increased dose of siderophore treatment in *A. nidulans* thus suggests dose-dependent increase in cell death. This further confirms that the siderophore is inducing cell lysis leading to membrane damage in *A. nidulans*. Similar fungicidal effect of a Blad-Containing Oligomer on fungi including *Candida* spp., *Cryptococcus neoformans*, and *Saccharomyces cerevisiae* has been observed where treatment of the polypeptide led to membrane damage as evident in PI staining (Pinheiro et al., 2016). In yet another study, destruction of plasma membrane in a similar manner has been related with cell death in plant fungal pathogen *Colletotrichum acutatum* by cinnamon essential oil (He et al., 2018).

Complete growth inhibition on exogenous supply of 54 μg/ml of catecholate siderophore and deterioration of fungal morphology at lower catecholate concentrations was hypothesized to occur due to chelation of all available iron by catecholate siderophore as well as absence of any compensatory mechanism creating an iron starved condition as mentioned earlier. To check this hypothesis, excess iron was added to the media along with catecholate siderophores. Evident growth was not restored; rather, an increase in iron uptake upon exogenous application of catecholate siderophore was observed. This indicated enhanced accumulation of iron in the presence of siderophore. Different strains of *Aspergillus* spp. other than *A. nidulans* have been reported to acquire iron from xenosiderophores (Wiebe and Winkelmann, 1975). Oberegger et al. (2001) have reported uptake of iron *via* the ferri-enterobactin complex in *A. nidulans.* Iron is well known to cause oxidative stress *via* Fenton reaction leading to protein and DNA damage (Yu et al., 2007). Also, certain *A. nidulans* mutants lacking ability of intracellular iron storage through siderophore ferricrocin have been observed to experience oxidative stress due to increased labile iron pool level (Eisendle et al., 2003). Thus, it was speculated that iron overaccumulation mediated by catecholate siderophores possibly resulted in increased oxidative stress leading to damage and alteration in fungal growth.

Increase in intracellular iron concentration has been correlated with increased production of ROS *via* Fenton reaction (Winterbourn, 1995). DCFDA, a membrane-permeable dye, is known to indicate ROS level. Upon entering the cells, it converts into its deacetylated form by the action of cellular esterases, and when this deacetylated form comes in contact with ROS, it forms a strong fluorescing molecule, 2′,7′-dichlorofluorescein (DCF). The intensity of DCF remains proportional to the amount of ROS present. Increase in fluorescence intensity with increasing siderophore concentration as observed in DCFDA staining in the present study indicates ROS accumulation within the cells and suggests increased ROS generation in a dose-dependent manner. Various stresses including metal stress are known to cause imbalance in ROS homeostasis. To mitigate such oxidative stress, organisms have developed a protective strategy involving different antioxidative enzymes such as SOD, CAT, and APX. The metalloenzyme SOD is known to catalyze breakdown of superoxides releasing H_2_O_2_, which is further broken down to water and oxygen by CAT and APX, relieving oxidative stress. At an initial concentration of 18 μg/ml, SOD activity remained the same while there was reduction in activity of CAT. This condition would lead to higher amounts of H_2_O_2_ within the cell as there is reduced conversion through CAT. In the presence of enhanced intracellular Fe^2+^, as indicated by AAS, H_2_O_2_ gives rise to hydroxyl ions and hydroxyl radicals by Fenton reaction (Fenton, 1894), leading to further enhancement of oxidative stress. Such oxidative stress mediated by iron-induced Fenton reaction and increased ROS level has been confirmed by DCFDA staining as mentioned above. This led to lipid peroxidation and membrane damage. Increase in MDA content at 18 μg/ml siderophore treatment supports free radical-induced membrane damage. Higher amount of siderophore treatment leads to greater intracellular Fe^2+^, which disturbs cellular homeostasis and an alteration of antioxidative enzyme activities occurs to counteract this effect. Enhancement in SOD activity at the highest siderophore treatment seems to be an attempt to minimize ROS, which, coupled with reduction in APX activity at 36 and 44 μg/ml, in turn increases the amount of H_2_O_2_. Also, augmented CAT activity is an indication of rise in the level of H_2_O_2_. This increase, however, seems inefficient to restore normal physiology. Increase in CAT and SOD activity attributed to H_2_O_2_ in response to cadmium metal stress has been reported in *A. nidulans* (Guelfi et al., 2003). In *Aspergillus oryzae*, difference in activity level has been observed under different metal exposures (Long et al., 2017). While Cu increased the activity of SOD and CAT similar to our report, Pb treatment led to decline in these two enzymes, which is, however, the case of APX in our study. Similar increase in CAT and SOD activity with subsequent decreased APX and POD activity has been observed in *Aspergillus tubingensis* MH189391, resulting in increased MDA content upon Sb metalloid treatment (Meghnous et al., 2019).

Interaction of MirA, MirB, and SreA supports present wet laboratory experiment results, suggesting the possibility of cellular iron accumulation *via* enterobactin–iron complex formation. *In silico* docking analysis was performed between Ent and MirA receptor of *A. nidulans* to reveal energy-based dynamics of possibility of xenosiderophore-mediated iron accumulation through fungal receptors as shown *in vitro* by Haas et al. (2003). Generated MirA model, with 14 major alpha helices and no β-sheets (Figure 5A) was similar to fungal TAFC transporter mirB (Raymond-Bouchard et al., 2012). MirA expression increases under condition of iron starvation (Haas et al., 2003), which might be the case here as Ent binds Fe with high affinity, creating an iron-deficient condition for the test organism (Harris et al., 1979; Karpishin and Raymond, 1992). Increased MirA expression might thus lead to increased iron uptake and accumulation through MirA *via* internalization of the Ent–Fe complex. It is evident from the docked structure that the Ent–Fe complex interacts and binds in a pocket-like binding site of MirA. Hydrophobic and pi-alkyl bond formation apparent in LigPlot analysis further confirmed the interaction. Thus, it can be deduced that a stable complex is formed between MirA of *A. nidulans* with exogenously supplied Ent complexed with Fe.

Fungi such as *Magnaporthe oryzae* are known to undergo ferroptotic cell death (Shen et al., 2020); however, there is no such report of ferroptosis in *A. nidulans* to the best of our knowledge. In the present study, introduction of xenosiderophore facilitates enhanced uptake of iron, possibly *via* MirA receptors. Excessive intracellular iron accumulation disturbs the physiological balance of ROS leading to oxidative stress. Antioxidative enzymatic machinery attempts to counteract this stress but is unable to do so beyond a threshold. One of the prime effects of increased free radical generation is membrane disruption that leads to loss of cell integrity. Thus, growth inhibition and cell death can be attributed to increased oxidative stress and greater damage to cellular membranes. Such membrane injury has been associated with ferroptosis, which occurs only upon iron accumulation leading to ROS accumulation and lipid peroxidation causing cell death (Dixon et al., 2012).

The present investigation establishes siderophore-induced hyperaccumulation of iron as a possible cause of ferroptotic death in *A*. *nidulans* and paves the way for testing efficacy of siderophores and their employment against other fungal pathogens.

## Data Availability Statement

The original contributions presented in the study are included in the article/supplementary material, further inquiries can be directed to the corresponding authors.

## Author Contributions

AK, PS, and ASr designed the experiment. AK performed the experiment. AK, ASr, ASa, and RKS analyzed the data. AK, ASr, ASa, PS, RK, UM, RR, and GA drafted the manuscript. All authors contributed to the article and approved the submitted version.

## Conflict of Interest

The authors declare that the research was conducted in the absence of any commercial or financial relationships that could be construed as a potential conflict of interest. The reviewer NT declared a shared affiliation with one of the authors, RR, to the handling editor at the time of review.

## Publisher’s Note

All claims expressed in this article are solely those of the authors and do not necessarily represent those of their affiliated organizations, or those of the publisher, the editors and the reviewers. Any product that may be evaluated in this article, or claim that may be made by its manufacturer, is not guaranteed or endorsed by the publisher.

## Footnotes

1
https://string-db.org/

2
https://cactus.nci.nih.gov/translate/
